# The Effect of Pre-Injury Anti-Platelet Therapy on the Development of Complications in Isolated Blunt Chest Wall Trauma: A Retrospective Study

**DOI:** 10.1371/journal.pone.0091284

**Published:** 2014-03-07

**Authors:** Ceri Battle, Hayley Hutchings, Omar Bouamra, Phillip A. Evans

**Affiliations:** 1 NISCHR Haemostasis Biomedical Research Unit. Morriston Hospital, Swansea, United Kingdom; 2 College of Medicine, Swansea University, Swansea, United Kingdom; 3 Trauma Audit and Research Network, University of Manchester, Manchester, United Kingdom; King’s College London School of Medicine, United Kingdom

## Abstract

**Introduction:**

The difficulties in the management of the blunt chest wall trauma patient in the Emergency Department due to the development of late complications are well recognised in the literature. Pre-injury anti-platelet therapy has been previously investigated as a risk factor for poor outcomes following traumatic head injury, but not in the blunt chest wall trauma patient cohort. The aim of this study was to investigate pre-injury anti-platelet therapy as a risk factor for the development of complications in the recovery phase following blunt chest wall trauma.

**Methods:**

A retrospective study was completed in which the medical notes were analysed of all blunt chest wall trauma patients presenting to a large trauma centre in Wales in 2012 and 2013. Using univariate and multivariable logistic regression analysis, pre-injury platelet therapy was investigated as a risk factor for the development of complications following blunt chest wall trauma. Previously identified risk factors were included in the analysis to address the influence of confounding.

**Results:**

A total of 1303 isolated blunt chest wall trauma patients presented to the ED in Morriston Hospital in 2012 and 2013 with complications recorded in 144 patients (11%). On multi-variable analysis, pre-injury anti-platelet therapy was found to be a significant risk factor for the development of complications following isolated blunt chest wall trauma (odds ratio: 16.9; 95% confidence intervals: 8.2–35.2). As in previous studies patient age, number of rib fractures, chronic lung disease and pre-injury anti-coagulant use were also found to be significant risk factors.

**Conclusions:**

Pre-injury anti-platelet therapy is being increasingly used as a first line treatment for a number of conditions and there is a concurrent increase in trauma in the elderly population. Pre-injury anti-platelet therapy should be considered as a risk factor for the development of complications by clinicians managing blunt chest wall trauma.

## Introduction

The use of anti-platelet therapy has increased over the last decade as a result of research which has reported their effectiveness in preventing cardiovascular events in high risk populations. [1] A simultaneous change has also occurred in trauma epidemiology, with an ever-increasing elderly trauma population. [2] Anti-platelet therapy is more common in the elderly age group primarily due to the higher incidence of co-morbidities. [3] The therapeutic mechanisms of anti-platelet agents include inhibition of platelet aggregation which results in the impairment of normal haemostasis. [4] Research has demonstrated that this impairment can lead to increased incidence of post-traumatic intracranial haemorrhage, potentially increasing morbidity and mortality in traumatic brain injury patients. [1], [3], [4] Limited research has been completed to date which investigates whether outcomes are also adversely affected by pre-injury anti-platelet therapy in the isolated blunt chest wall trauma population.

Blunt chest wall trauma accounts for over 15% of all trauma admissions to Emergency Departments worldwide. [5] Reported mortality is as high as 22% in this patient cohort. [6] The difficulties in the management of the blunt chest wall trauma patient are becoming increasingly well recognised in the literature. [7], [8] The blunt chest wall trauma patient commonly presents to the Emergency Department (ED) initially with no respiratory difficulties, but can develop respiratory complications approximately 48 to 72 hours later. [9], [10] Clinical symptoms are not considered an accurate predictor of outcome following non-life threatening blunt chest wall trauma. [11] A number of well-documented risk factors for morbidity and mortality exist for blunt chest wall trauma including patient age, pre-existing disease, number of ribs fractured, pre-injury anticoagulant use and the on-set of pneumonia during the recovery phase. [12], [13]The aim of this study was to investigate whether the use of pre-injury anti-platelets is a risk factor for the development of complications in blunt chest wall trauma patients.

## Methodology

### Setting

A retrospective study design was used in order to examine the medical notes of all blunt chest wall trauma patients who presented to the ED of a large regional trauma centre in South Wales (Morriston Hospital) in 2012 and 2013. Morriston hospital has approximately 90,000 presentations to the ED per year and serves a population of 450,000 people. Those patients coded as ‘blunt chest trauma’ or ‘rib fractures’ were identified using the hospital database. Patients with any significant concurrent injuries were excluded to reduce the effect of confounding.

### Sample

We wished to include sufficient patients that we could present the unadjusted and adjusted odds ratios and 95% confidence intervals for the risk factors for the development of complications following blunt chest wall trauma. Peduzzi et al (1995) suggested that the number of patients needed to ensure sufficient power in a retrospective cohort study is equivalent to ten events per variable (EPV) being investigated. [14] In this study we set out to investigate six variables or risk factors based on previous research therefore a minimum of 60 events (on-set of complications following blunt chest wall trauma) were required.

### Data Collection

The medical notes were reviewed following guidelines suggested in a study by Gilbert et al (1996). [15] The ED medical notes of all patients aged 16 years and over presenting to the ED of Morriston Hospital in 2012 and 2013 were examined and data recorded on a pre-designed database. A validation check was completed in which an additional researcher checked the accuracy of data input for 10% of all patients, in order to reduce information bias. If a patient’s notes had missing or incomplete data for the variables under investigation, they were still included in the database.

The dataset included demographic variables such as age, gender and injury mechanism. The independent variables examined were defined a priori and consisted of the risk factors for mortality and morbidity highlighted previously in the literature. [13] These included patient age, chronic lung disease, number of rib fractures and pre-injury anticoagulant use. Pre-injury anti-platelet use was also investigated as the primary variable of interest in this study. The outcome measure investigated was the development of complications in the recovery phase following blunt chest wall trauma. To ensure confidentiality, patients’ names were not recorded during the data collection period. The dataset was also stored on a hospital encrypted computer to ensure data security (Safe-end protector encryption).

### Ethics Statement

The South West Wales Research and Ethics Committee confirmed that ethical approval was not required for this study and also waived the need to obtain written informed consent from the patients. All patient information was anonymised and de-identified prior to analysis.

### Definition of Variables

The patient’s age, chronic lung disease including; chronic obstructive pulmonary disease or bronchiectasis, pre-injury anti-coagulant use (all anti-coagulants and any dose included), pre-injury anti-platelet use (all anti-platelets and any dose included) were all identified from the medical notes. The number of rib fractures was determined by the clinical notes however in the cases where the number of rib fractures could not be determined using the clinical records, then the x-ray report (IMPAX software) was reviewed by the investigators.

The development of complications during the recovery phase following blunt chest wall trauma was the composite outcome measure investigated in this study. Data collection for this outcome was completed from the time the patient presented to the ED through to discharge from hospital. Patients were reported to have developed complications if one or more of the following were documented in their medical records; in-hospital mortality, morbidity including all pulmonary complications (chest infection, pneumonia, haemothorax, pneumothorax, pleural effusion, or empyema), ICU admission, an unplanned representation to the ED, or a prolonged length of stay as defined as a total hospital stay of seven or more days.

### Data Analysis

Baseline characteristics were presented as median and interquartile range (due to non-normal distributions) for the continuous variables and numbers and percentages for categorical variables. Differences between the baseline characteristics were analysed using Mann Whitney U test (continuous variables) and Fisher’s Exact test (categorical variables). Odds ratios and 95% confidence intervals were presented from the univariable analysis. All significant prognostic variables at 5% significance on the univariable analysis were included in final analysis. Multivariable logistic regression analysis using fractional polynomials (to assess linearity of the continuous variables) was used to identify significant predictors using the Likelihood test statistic. There was less than 2% missing data therefore we used a simple imputation of the mean method to avoid exclusion of patients from the final analysis. [16] Stata Release 13 was used for all data analysis.

## Results

A total of 1303 isolated blunt chest wall trauma patients presented to the ED in Morriston Hospital in 2012 and 2013 with complications recorded in 144 patients (11%). Data including demographics, independent and dependent variables were recorded for each of the patients. With the exception of recording of eleven patients’ oxygen saturations, there were no missing variables in the dataset. Pre-injury anti-platelet therapy was recorded in 116 patients (9%) of the entire cohort. Aspirin was most commonly used antiplatelet medication (104 patients, 8%), followed by NSAIDs (six patients, 0.5%), clopidogrel (five patients, 0.4%) and combined aspirin and clopidogrel (one patient, 0.08%). Warfarin was the most commonly used anti-coagulant (n = 35, 3%) followed by Dabigatran (two patients, 0.2%). Haemorrhagic events (haemothorax) were recorded in 36 out of 144 (25%) patients who developed complications. Table 1 highlights the demographic data for each of the patients. Patients’ gender, injury mechanism and complication rate are presented.

**Table 1:** Patients’ demographics, injury mechanisms and complication rate.

	Total patients (n = 1303)
Age (mean/SD)	50 (±21)
**Gender**	
Male	761 (58%)
Female	542 (42%)
**Injury Mechanism**	
Fall	907 (70%)
Road traffic accident	182 (14%)
Assault	89 (7%)
Sporting injury	105 (8%)
Other	20 (2%)
**Complication**	144 (11%)
Mortality	5 (0.4%)
Morbidity	56 (4%)
ICU admission	16 (1%)
Unplanned representation to the ED	22 (2%)
Prolonged length of stay	57 (4%)

n: number, SD: standard deviation, ICU: Intensive Care Unit, ED: Emergency Department.

Table 2 highlights the results of each risk factor investigated using univariate analysis. The unadjusted odds ratios and their 95% confidence intervals are presented for each categorical risk factor investigated. The risk factors for development of complications following blunt chest wall trauma were patient age, number of rib fractures, chronic lung disease, pre-injury anti-coagulant use and pre-injury anti-platelet use.

**Table 2:** Results of univariate analysis: Risk factors for complications in blunt chest wall trauma.

	Complicationsn = 144 (11%)	No complications n = 1159 (89%)		
Categorical variables	n (%)	n (%)	p value	Unadjusted OR (95%CI)
Chronic lung disease	17	27	p<0.001	5.6 (3.0–10.6)
Pre-injury anti-coagulant use	21	16	p<0.001	12.2 (6.2–24.0)
Pre-injury anti-platelet use	62	54	p<0.001	15.5 (10.1–23.7)
**Continuous variables**	**Median (IQR)**	**Median (IQR)**	**p value**	
Age	70 (26)	46 (31)	p<0.001	
Number of rib fractures	2 (2)	0 (0)	p<0.001	

OR: odds ratio; CI: confidence interval. Fisher Exact tests were used to analyse categorical variables and Mann-Whitney test for the continuous variables.

No significant association was found between the variables pre-injury anti-coagulants and pre-injury anti-platelets. The only significant associations found were between anticoagulant use and chronic lung disease (p = 0.001), and antiplatelet use and chronic lung disease (p<0.001). However this association led to no significant interactions in the model on multivariate analysis (p = 0.77 and p = 0.78 respectively). Results of the multivariable logistic regression analysis are outlined in Table 3 and the adjusted odds ratios and the 95% confidence intervals presented. Results highlight that the significant risk factors for the development of complications following blunt chest wall trauma were age, number of rib fractures, chronic lung disease, pre-injury anticoagulant use and pre-injury anti-platelet use. The final model omnibus test was p<0.001 indicating an accurate overall model for predicting complications with the predictors investigated.

**Table 3:** Risk factors and their adjusted odds ratios for the development of complications following blunt chest wall trauma (all p<0.05).

Risk factor	Complications Adjusted OR (95%CI)	p value
Age	1.0 (1.0–1.0)	p = 0.017
Chronic lung disease	5.6 (2.2–14.6)	p<0.001
Number of rib fractures	5.0 (3.9–6.5)	p<0.001
Pre-injury anti-coagulant use	22.0 (8.3–58.2)	p<0.001
Pre-injury anti-platelet use	17.8 (8.6–37.0)	p<0.001

OR: odds ratios; CI: confidence intervals. OR for age is per one year increase. OR for number of rib fractures is per one fracture increase.

## Discussion

As no current guidelines exist for the management of this patient group, recognition of the high risk patient in the ED is not always straightforward due to the nature of the injury and its recovery phase. The blunt chest wall trauma patient who walks into the ED with no immediate life-threatening injury will commonly develop complications up to 72 hours or more post injury, which may also prove life-threatening. [9], [10] An understanding of the risk factors for development of late complications in blunt chest wall trauma patient could assist in the accurate risk stratification of this patient group in the ED and thus improve outcomes. A number of risk factors for the development of complications following blunt chest trauma have been investigated in this single centre retrospective study including patient age, number of rib fractures, chronic lung disease and pre-injury anti-coagulant use. These results support the reported findings of previous studies.[9], [12], [13], [17]–[19] The interesting finding in this study however is that pre-injury anti-platelet use is also a significant risk factor for complications following blunt chest wall trauma.

The antithrombotic action of aspirin (acetylsalicylic acid) is due to inhibition of platelet function by acetylation of the platelet cyclooxygenase (COX) at the functionally important amino acid serine_529_. This results in an irreversible inhibition of platelet-dependent thromboxane formation. [20], [21] It is well-recognised that aspirin is considered the gold standard antiplatelet agent for prevention of arterial thromboses. [21] The optimum dose of aspirin as an antithrombotic drug can differ in different organ circulations. While 100 mg/day is sufficient for prevention of thrombus formation in the coronary circulation, higher doses may be required for the prevention of vascular events in the cerebral and peripheral circulation. [20] Any effective dose of aspirin however is associated with an increased risk of bleeding, especially in the trauma population [20].

Over the last decade, the use of antiplatelet therapy has increased considerably, as a result of national and international guidelines promoting their widespread in high-risk populations and particularly in the elderly. [3] The epidemiology of the trauma population has concurrently changed, with an ever increasing prevalence of older age-groups. [3] It is in this increasing elderly population where antiplatelet drug use is more prevalent. As a result of this increased use of anti-platelet therapy as the population ages, it is vital that trauma clinicians understand the risks involved with their use. Pre-injury anti-platelet use has been increasingly investigated as a risk factor for various poor outcomes in traumatic head injuries. [1], [3], [4] A number of studies have concluded that anti-platelet therapy is a risk factor for both short-term and long-term unfavourable outcomes in subjects with head injury, with increased risk of death, permanent vegetative state and severe disability. [3] A further difficulty currently facing trauma clinicians is that limited research exists that addresses the efficacy or utility of reversing pre-injury anti-platelets therapy in traumatic head injury. [1].

Another difficulty facing emergency physicians in the management of trauma patients using pre-injury anti-platelets is the variation between patients in their response to the medication. Platelet function tests can be carried out on trauma patients in order to assess number and size of platelets and also platelet haemostatic function. The type of tests completed, specifically either global screening tests or specific assays will depend on the testing equipment available at that emergency department. The decision as to whether the blunt poly-trauma patient requires platelet transfusion therefore is dependent on a number of factors. In isolated blunt chest trauma however platelet function may not routinely be considered by the emergency physician even though delayed haemothorax is not an uncommon entity in this patient group. [10] It could be suggested that tests for platelet function may be an important consideration for the emergency physician managing the isolated blunt chest trauma patient however further research is required to support this suggestion.

To date, no research exists investigating the effect of pre-injury anti-platelet use in blunt chest trauma. Pulmonary contusion occurs in 25–35% of all blunt chest traumas and is defined as an injury to the alveolar capillaries, in the absence of a lung laceration. [22] This results in accumulation of blood and other fluids within the lung tissue which subsequently interferes with gas exchange leading to hypoxia. [22] If blood and plasma are leaked into the alveoli in rib fractures patients, it possible that this pathophysiological process is exacerbated in patients receiving pre-injury anti-platelet therapy. This variable has not been investigated to date as a risk factor for poor outcomes following blunt chest wall trauma. In one previous study, pre-injury anti-platelet therapy was reported to reduce the incidence of acute lung injury in a medical ICU population, of which there were 24 poly-trauma patients in the study cohort and only eight of these were using pre-injury anti-platelets. [23] The authors of this study also do not state whether these trauma patients had sustained head injuries and therefore comparison between the studies is not possible [23].

In a recent study by Harr et al (2013), the use of pre-injury anti-platelet therapy was reported to be associated with a decreased risk of lung dysfunction in high risk blunt trauma patients who receive blood transfusions. [24] Neal et al (2014) reported similar findings, concluding that NSAID use before admission for severe injury is associated with a reduced incidence of trauma-induced coagulopathy. [25] The results of this study do not support the findings of Harr et al (2013) and Neal et al (2014) however the study populations are have distinct differences. The patients included in the study by Harr et al [2013] were all severely injured poly-trauma patients who had all received blood transfusions and were at considered at risk of multi-organ failure. [24] Similarly the poly-trauma patients in the study by Neal et al (2014) all presented with haemorrhagic shock following blunt injury. [25] In contrast, the patients in our study were isolated blunt chest trauma patients who were not in haemorrhagic shock and had not received blood transfusions. Patients with concurrent injuries were excluded. Pathophysiological mechanisms of isolated blunt chest trauma are evidently very different to those of major poly-trauma. It is not possible therefore to compare these studies findings due to the heterogeneity in study populations.

### Study Limitations

One potential limitation of the study is that polytrauma patients were excluded so the results are only generalisable to isolated blunt chest wall trauma patients. The number of haemorrhagic complications was recorded on data collection however data were not collected regarding thromboembolic events in the study population and this information may have been beneficial to the clinician. Similarly the very small number of patients taking anti-platelet therapy other than aspirin precludes analysis into which therapy is most associated with specific complications. Lack of data regarding platelet function in individual patients and platelet transfusions may have influenced the study results.

As a result of the study design and the inherent nature of patients, a number of the independent variables investigated were potentially interdependent and an increase in one variable inadvertently leads to an increase in another. Multivariable logistic regression with backward elimination techniques was used in an attempt to address this issue of collinearity. It is also possible that a confounding variable that influences the results was not considered in the data collection or analysis. It could therefore be suggested that the discovered association between anti-platelet therapy and chest wall complications is the result of both risk factor and outcome being related to common underlying unmeasured pathologies of some kind. In prognostic clinical research however this is difficult to overcome due to the nature of the study population and therefore the results of the study should be interpreted with this in mind.

The use of the database to identify the patients for inclusion in this study may have resulted in a degree of selection bias. Errors may have occurred in the collation of the list of patients from the hospital database and similarly by the doctors completing the coding form in the ED. Similarly, as the data were being collected for each of the patients from their ED medical notes, reliance was placed on the information being both accurately and legibly documented. This may have led to some error in data collection and should be considered when interpreting the study results. The most appropriate method of overcoming a number of the study limitations is to complete a prospective study.

## Conclusions

This study has highlighted a number of well-recognised risk factors for the development of complications following blunt chest wall trauma. These included patient age, number of rib fractures, chronic lung disease and pre-injury anti-coagulant use. This is the first study in which pre-injury anti-platelet use has been reported as a risk factor for complications following isolated blunt chest wall trauma. Pathophysiological mechanisms differ substantially between blunt poly-trauma patients suffering trauma-induced coagulopathy and isolated blunt chest trauma patients and as a result, comparisons between such studies are not appropriate. The results of this study are therefore distinctive from any previous research and should be considered by emergency physicians managing isolated blunt chest trauma.

Anti-platelet therapy is now a well-recognised risk factor for potential poor outcomes following severe traumatic head injuries and trauma clinicians are becoming increasingly aware of its negative impact in the elderly trauma patient. Despite the limitations associated with a single centre retrospective study, this results of this study have highlighted an important clinical finding that trauma clinicians should consider when managing the blunt chest wall trauma patient in the ED. A prospective multi-centre study is planned to further investigate the impact of pre-injury anti-platelet use on outcomes in blunt chest wall trauma patients.
